# Short ramus reconstruction for hemi-mandibulectomy defect: Case reports

**DOI:** 10.1016/j.jpra.2022.06.009

**Published:** 2022-07-05

**Authors:** Eiji Hirai, Shunji Sarukawa, Jinsil Park, Seiko Fujii, Takeshi Nishikawa, Kozo Yamamoto

**Affiliations:** a Department of Oral and Maxillofacial Surgery, Oita Red Cross Hospital, Japan; b Department of Plastic Surgery, Saitama Medical University, International medical center, Japan; c Department of Oral and Maxillofacial Surgery, Shin-Yurigaoka General Hospital, Japan

**Keywords:** Mandible, Mouth neoplasm, Reconstructive surgical procedures, Condyle

## Abstract

Reconstruction of the mandible following hemimandibulectomy is difficult and complex.

The appropriate approach to condylar reconstruction remains controversial. In this report, the authors propose the concept of “short ramus reconstruction” after hemimandibulectomy. In this technique, a neocondyle is constructed around the base of the condyle to avoid trismus and ankylosis. Four patients underwent short condylar reconstruction using fibula free flaps. Post-surgery, no patient developed trismus or ankylosis. Centric occlusion, good masticatory function, and favourable aesthetic outcomes were achieved in all cases. “Short ramus reconstruction” is a simple and convenient method to reconstruct the mandible following hemimandibulectomy.

## Introduction

Restoration of mandibular contour and function are the goals of reconstruction. Condylar loss may lead to impaired jaw opening, mastication, deglutition, and speech.^1^ Reconstruction of the mandible following resection is difficult and complex but is essential for achievement of good functional and aesthetic outcomes, especially in the case of a large resection that includes the condyle.^2^ Appropriate management of the condyle following a large mandibular resection involving the condyle remains controversial.^1^^,^^3^ Several potential complications are associated with condylar reconstruction, including skull base erosion, ankylosis and temporomandibular joint (TMJ) dysfunction, and increased difficulty to recapitulate the TMJ with high fidelity.^1^

In this article, a novel concept for reconstructing the ramus, namely “the short ramus reconstruction,” developed to overcome the risks of erosion, ankylosis, and trismus, is described.

## Materials and methods

### Surgical procedures

To assess the bony defect in each patient, a CT scan was obtained, and a 3-dimensional stereolithographic model was produced. The model was used as a guide to pre-contour a reconstruction plate, and a silicon rubber template was used to replicate the fibula.

**Figure 1:**
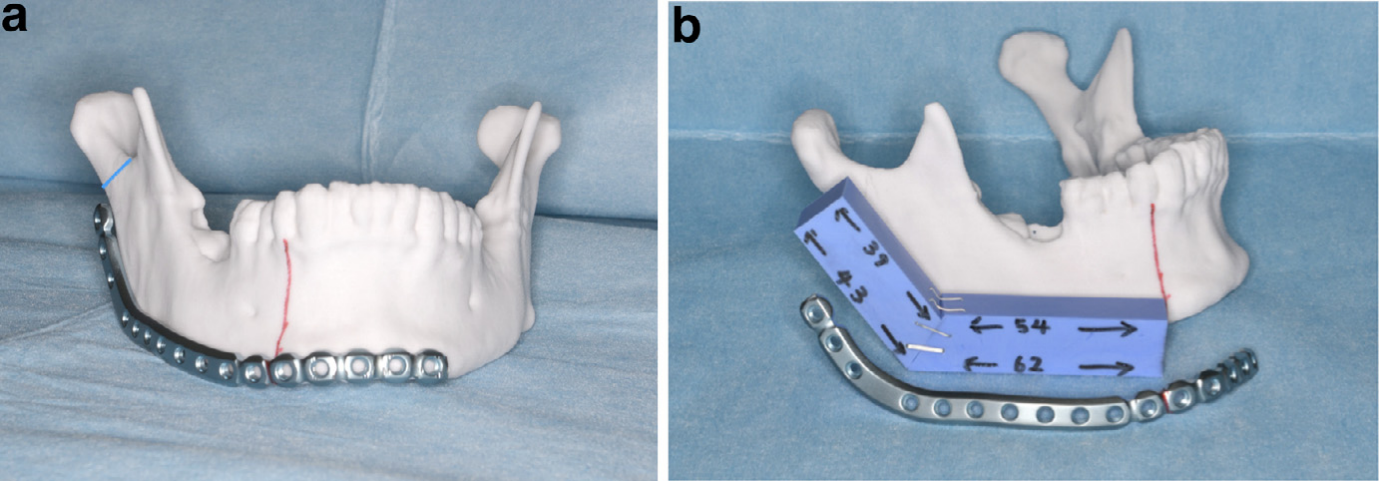
Patient 2 Photograph depicting presurgical planning/prototyping modelling. (A) The reconstruction plate was contoured and placed at the inferior border of the mandible. Blue line is mark of placement distal end of fibula. (B) Silicon paste was used to create an ideal replica of the reconstructed fibula; the distal end of the fibula was positioned around the base of the condyle.

A prosthesis was not used to reconstruct the condyle and the distal end of the fibula free flap was not placed in the precise condylar position, but rather around the base of condyle. (Fig. 1) The distal bone edge of the fibula graft was not enclosed by periosteum or rounded. No suture was used to suspend the neocondyle.

Intermaxillary fixation (IMF) was unnecessary in some cases. IMF, using inter-arch elastics for 2 weeks, was only applied when the patient was unable to independently achieve centric occlusion postoperatively.

### Patients

The present study was approved by the Ethics Committee of Oita Red Cross hospital (251). Written patient consents were obtained from all the study participants. Four patients were evaluated retrospectively. Each underwent hemimandibulectomy and reconstructive surgery, using vascularized fibula transplantation, between February 2015 and August 2019. The patient chart was reviewed for each case to source information relating to the patient's age, aetiology of the mandibular defect, maximum interincisal opening, occlusion, and diet (Table 1).

**Table 1:** Patient characteristics and results.

Patient	Age	Cause of Mandibular defect	Graft	Adjuvant Therapy	IMF	MIO (mm)	Diet	Occulusion	Follow up period (months)
1	65	Malignancy	Fibula	–	none	50	Regular	Centric	78
2	52	Malignancy	Fibula	–	2weeks	48	Reular	Centric	52
3	73	Malignancy	Fibula	–	none	40	Regular	Centric	21
4	54	Benign tumour	Double-barrelled fibula	–	2weeks	42	Regular	Centric	24

## Results

Follow up was between 18 and 78 months for four patients. (Median: 38 months) The patients were followed up at 8 and 36 months.

Healing progressed uneventfully in all cases, excluding one. In this case the reconstruction plate fractured 3 months postoperatively and additional surgery was required to reposition and fix the bone graft using a mini plate.

Postoperatively, all patients achieved centric occlusion, a mean maximum interincisal opening of 45 mm (range: 40–50 mm) and good masticatory function, with the ability to chew solid foods without any dietary restriction. Trismus did not present in any case.

## Patient presentation

### Patient 2

A 53-year-old patient was diagnosed with squamous cell carcinoma of the right lower alveolus (T4aN0) and underwent right hemimandibulectomy and ipsilateral supraomohyoid neck dissection, followed by immediate mandibular reconstruction using a vascularized fibula osteocutaneous flap. The oral lining was covered by a skin paddle.

Before surgery, prototyping modelling was performed. The reconstruction plate was contoured and a silicon rubber replicate of the graft was created to reconstruct the mandible. The neocondyle was positioned equivalent to the base of condyle, creating a short ramus (Fig. 1). During surgery, care was taken to position the lower edge of the transplanted fibula and the residual mandible according to the placement achieved during presurgical modelling.

**Figure 2:**
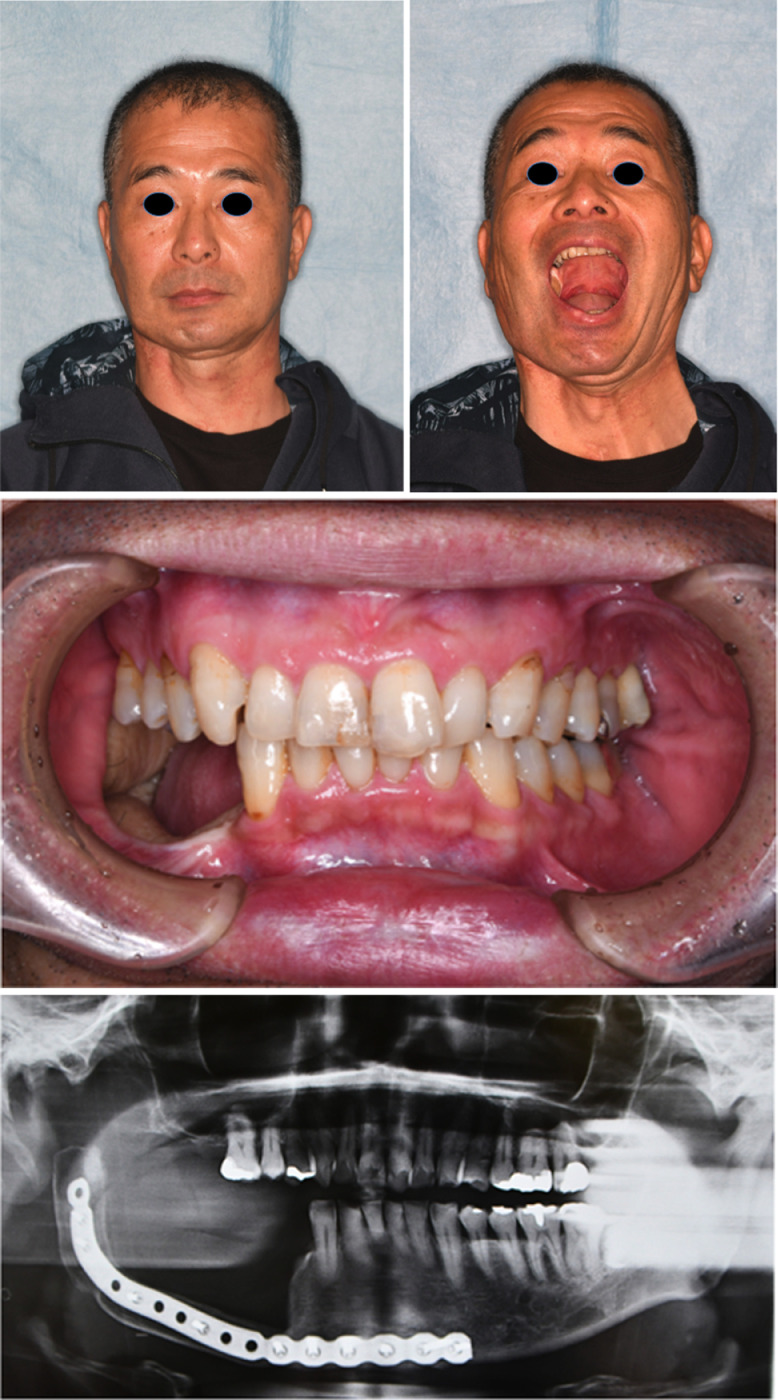
Patient 2—48 months post-surgery. (A) Extraoral postoperative photograph showing the anterior view. (B) Extraoral postoperative photograph showing the anterior view with mouth open. (C) Intraoral photograph showing good postoperative occlusion. (D) Postoperative panoramic radiograph. The distal end of fibula is seen to be located at the base of the condyle.

The postoperative course was uneventful, and no adjuvant therapy was provided. Thirty-six months after the initial surgery, the tumour had not recurred, centric occlusion had been maintained, no signs or symptoms of trismus presented, and the patient followed an unrestricted diet (Fig. 2).

## Discussion

The goal of mandibular reconstruction is to restore masticatory function and aesthetics. TMJ dysfunction following tumour resection of the hemimandible is a frequent sequela of condylar head reconstruction. Numerous approaches have been attempted; replacement of the condyle and reconstruction of the TMJ is usually disappointing.^4^

Several authors have suggested that soft tissue free flap reconstruction alone may be adequate for reconstruction of segmental posterior mandibular defects.^5^^,^^6^ However, Hanasono et al.^7^ have reported a number of drawbacks associated with soft tissue free flap reconstruction that are not typically evident when using vascularized bone flap reconstruction. These drawbacks include the inability to perform osseointegrated dental restoration of the posterior mandibular region and deviation of the mandible towards the resected side. It should also be noted that when the resected condyle was reconstructed using a vascularized bone flap, malocclusion occurred less frequently than that observed when using soft tissue flap reconstruction.^7^

Numerous papers recommend that bony reconstruction of a TMJ defect should be as precise as possible, with the neocondyle set in the glenoid fossa.^1^^,^^3^^,^^7^, ^8^, ^9^ Various methods were described to prevent ankylosis of the joint, including preservation of the disc, suspension of the neocondyle, and the muscle insert technique.^7^^,^^9^

Neocondylar movement is an important concept. Akashi et al. evaluated the movement of neocondyles constructed with fibula free flaps using four-dimensional computed tomography (4DCT). In one patient who underwent hemimandibulectomy and fibula free flap reconstruction, the most cranial portion of the fibula flap did not protrude forward to the articular eminence.^10^ Therefore, it can be concluded that a neocondyle when positioned in the glenoid fossa has the potential to interfere with jaw movement and mouth opening, and heighten the risk of trismus.

Restoration of the mandibular angle and ramus is important for attainment of a favourable cosmetic result. Soft tissue reconstruction for hemimandibulectomy tends to blunt the mandibular angle and a loss of tissue volume may occur on the reconstructed side.^7^ The final contour of the mandible obtained following reconstruction using a soft tissue flap is less predictable than that achieved after bony reconstruction.

**Figure 3:**
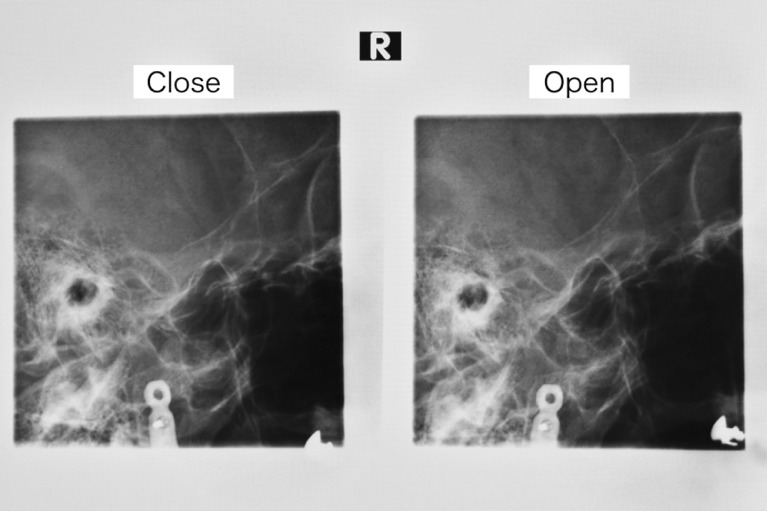
**Patient 2**—Temporomandibular joint radiograph. The distal end of the fibula is separated from the glenoid fossa.

Consequently, in terms of function and aesthetics, vascularized bone reconstruction is considered necessary for a hemimandibulectomy defect, while positioning of the neocondyle in the glenoid fossa is regarded as unnecessary. Based, on these observations, the concept of “short ramus reconstruction” for hemimandibulectomy was conceived and proposed. Positioning of the neocondyle have a possibility of dysfunction, so in this novel technique there was no condylar head, no suspension and no seat in the glenoid fossa. In the present case, radiographic examination of the TMJ in open mouth position, following “short ramus reconstruction,” demonstrated a separation between the neocondyle and the glenoid fossa (Fig. 3). The other results were the same. Centric occlusion, no trismus, and well-maintained facial contour were achieved in all cases.

In conclusion, reconstruction of the mandibular condyle in its precise location following hemimandibulectomy is difficult and risk of postoperative TMJ dysfunction is high. Conversely, “short ramus reconstruction” is relatively easy and associated with a low risk of dysfunction.

## Role of funding source

This research did not receive any specific grant from funding agencies in the public, commercial, or not-for-profit sectors.

## Ethical approval

Approved by Oita Red Cross Hospital ethics committee No. 251.

## Patient consent for photo publication

Written permission obtained.

## Declaration of Competing Interest

None.
